# Sanya climatic-treatment cohort profile: objectives, design, and baseline characteristics

**DOI:** 10.3389/fpubh.2023.1290303

**Published:** 2023-10-19

**Authors:** Haidao Guan, Guiyan Yang, Jiashi Gao, Xiaoya Lin, Chao Liu, Han Ren, Duyue Chen, Lingyao Zhou, Qian Hu, Yongzhen Huang, Yumei Zhao, Shilu Tong, Zhaohui Lu, Shijian Liu, Dan Wang

**Affiliations:** ^1^Department of Science and Education, Hainan Branch, Shanghai Children's Medical Center, School of Medicine, Shanghai Jiao Tong University, Sanya, China; ^2^Department of Hospital Management, Hainan Branch, Shanghai Children's Medical Center, School of Medicine, Shanghai Jiao Tong University, Sanya, China; ^3^Department of Hospital Infection, Hainan Branch, Shanghai Children's Medical Center, School of Medicine, Shanghai Jiao Tong University, Sanya, China; ^4^Department of Nursing, Hainan Branch, Shanghai Children's Medical Center, School of Medicine, Shanghai Jiao Tong University, Sanya, China; ^5^Department of Clinical Epidemiology and Biostatistics, Children Health Advocacy Institute, School of Medicine, Shanghai Jiao Tong University, Shanghai, China; ^6^School of Public Health and Social Work, Queensland University of Technology, Brisbane, QLD, Australia; ^7^Department of Pediatric Surgery, Hainan Branch, Shanghai Children's Medical Center, School of Medicine, Shanghai Jiao Tong University, Sanya, China; ^8^Department of Big Center, Hainan Branch, Shanghai Children's Medical Center, School of Medicine, Shanghai Jiao Tong University, Sanya, China

**Keywords:** tropical climate, environmental factors, migratory people, asthma, allergic diseases

## Abstract

**Background:**

The prevalence of allergic diseases has increased globally, climate and environment also have important effects on respiratory or allergic diseases. However, population-based studies investigating the impact of tropical climates and environments on migratory-bird old people (MBOP) are lacking.

**Methods/Design:**

For this prospective cohort study, we recruited 756 participants from the community in Sanya City, Hainan Province, China. In addition to the completed baseline survey, a follow-up survey will be conducted during the periods of October–December and March–April for the next 3 years of MBEPs from northern China who spend the winter in Sanya. We will continue to record the height, weight, and blood pressure of all participants, as well as lung function for those with asthma and chronic obstructive pulmonary disease (COPD). Venous blood at baseline and urine samples will be collected during follow-up.

**Results:**

A total of 756 volunteers were recruited. Their average age is 66.1 years; 32.1% of them have high-school educations, while 37.3% have graduated from college or done undergraduate studies. The top five diseases in this cohort are allergic rhinitis (57.9%); eczema, urticaria, or dermatitis (35.6%); bronchitis and bronchiectasis (35.6%); asthma (14.7%); and emphysema (11.7%). Compared with their symptoms while at their summer places of residence, rates of remission reported by participants while living in Sanya were 80.4% for allergic rhinitis, 82.3% for bronchitis and emphysema, 85.2% for asthma, 96.0% for COPD (*P* < 0.001).

**Conclusions:**

The baseline survey has been completed. The preliminary findings support that a tropical climate may relieve the symptoms of allergic diseases in migratory-bird old people.

## Introduction

The prevalence of allergic diseases has increased globally in recent decades (1, 2). According to the World Health Organization's (WHO) 2019 report, approximately 262 million people worldwide suffered from asthma at the time, resulting in 455,000 deaths. Allergic diseases, including allergic rhinitis and eczema, together constitute a major global public-health burden (3). China is also facing this issue: according to epidemiological surveys, the prevalence of allergic diseases (4, 5) such as asthma (6, 7), allergic rhinitis (8), and eczema are increasing in China. Influencing factors include climate change (9), air pollution (10), environmental temperature (11), meteorological factors (12), and allergens (13).

Current studies on allergic diseases mainly focus on the effects of climate and environmental factors. There are reports on the treatment and relief of asthma in alpine environments (14, 15). However, population-based research is lacking on the effect of tropical climate and environment on migratory-bird old people (MBOP). Sanya, China has a unique tropical climate, with a minimum temperature of >15°C in winter; by contrast, the minimum winter temperature in northern China is below −40°C (16). Therefore, many MBEPs relocate from northern China to Sanya for the winter. It is reported that more than 1 million people spend the winter in Sanya every year (17).

In addition to drug treatment, climate, and environment also have important effects on respiratory or allergic diseases. In Europe, cave therapy is widely used to treat chronic airway diseases. Some studies have shown that exercise in winter combined with cave therapy can improve the quality of life (QoL) and allergic symptoms of adults with allergic rhinitis and/or asthma (18, 19). A systematic review showed that high-altitude climate therapy improved the lung function of adult asthma patients (15, 20). However, there is no relevant evidence that symptoms and climate-related factors of allergic diseases improve in MBEPs who move from high latitudes to low ones. The establishment of this cohort will help us better study the effect of Sanya's tropical climate on respiratory or allergic diseases, clarify risk factors related to these diseases, and determine whether such a climate effectively mitigates these diseases in MBEPs.

## Cohort description

### Study design, setting, and participants

**Figure 1:**
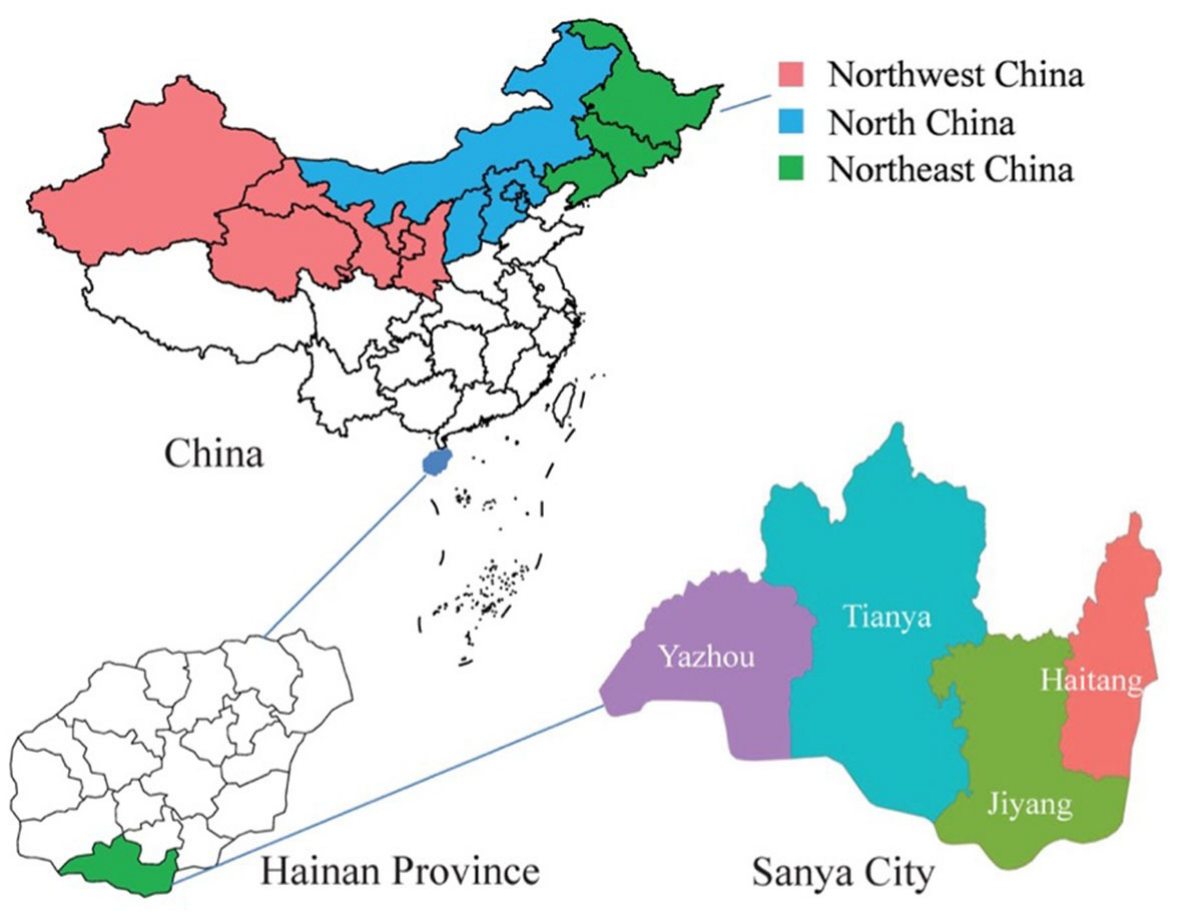
Geographical location of the Sanya migratory cohort. The shaded regions represent participants' original places of residence, red indicates the northwest China, blue represents the north China, and green signifies the northeast China.

This is a prospective cohort study whose subjects were recruited from the community in Sanya City, Hainan, the southernmost province in China from 2022 to 2025. We will conduct a follow-up survey focused on allergic diseases in MBEPs from northern China who move to Sanya for the winter (Figure 1). Northern China includes three northeastern provinces (Heilongjiang, Jilin, and Liaoning), five northern provinces (Beijing, Tianjin, Hebei, Shanxi, and the Inner Mongolia Autonomous Region), and five northwestern provinces (Xinjiang Uygur Autonomous Region; Ningxia Hui Autonomous Region; and Qinghai, Gansu, and Shaanxi Provinces). The subjects of this study are MBEPs who travel from northern China to Sanya in autumn, stay for the winter, and return to northern China in spring. The sample size was estimated by forced expiratory volume in 1 s (FEV_1_) (15), which was 92.8% ± 23.1 and 86.5 ± 26.2 for the trial and control groups, respectively. A type I error α = 0.05, β = 0.10, and loss of follow-up rate of 20% for 3 years, requiring 775 participants.

For easier follow-up, we recruited participants from communities of mainly MBEPs, who own apartments in Sanya and spend the winter there for many years. Inclusion criteria were as follows: (a) age 50–80 years; (b) suffering from allergic rhinitis, asthma, eczema, urticaria, or chronic obstructive pulmonary disease (COPD); (c) previous or winter residence in northern China; (d) willingness to participate in the study and be followed up on at the designated location for the subsequent 3 years; and (e) no difficulty in communication. Exclusion criteria were (a) other respiratory diseases and (b) communication barriers or unwillingness to cooperate with the requirements of the study.

Diagnosis of allergic rhinitis, asthma, eczema, urticaria, or COPD was based on self-reported disease history in face-to-face interviews at recruitment. Allergic rhinitis was defined as an affirmative response to the question “Do you have any nasal allergies, including hay fever?” according to the Allergic Rhinitis and Its Impact on Asthma guidelines (8). Asthma was defined as a self-reported history of asthma, diagnosis by a physician, or wheezing during the preceding 12 months (6). Eczema or urticaria was defined as self-reported history and/or diagnosis by a physician (21, 22). Atopic dermatitis was diagnosed by an affirmative response to the question “Have you had an itchy rash at any time in the past 12 months?” (8). COPD was defined as post-bronchodilator FEV_1_/forced vital capacity (FVC) < 0.7 according to the 2017 Global Initiative for Chronic Obstructive Lung Disease guidelines (23). Chronic bronchitis was defined as self-reported phlegm production for at least 3 months each year over 3 successive years (24).

## Data collection

### Demography and outcomes

A paper questionnaire was used to collect a range of basic information in march 2022. After obtaining informed consent, trained investigators collected relevant information in face-to-face interviews. The questionnaire covered demographic data, socioeconomic status, clinical disease characteristics, living habits, and past medical history (Table 1). Participants with asthma and COPD were evaluated using the Asthma Quality of Life Questionnaire (AQLQ) (25) and Saint George Respiratory Questionnaire (SGRQ) (26), respectively; SGRQ assesses the quality of life of participants with COPD. The primary outcome was the effect of Sanya's tropical climate on MBEPs with allergic or respiratory diseases, including symptoms evaluated by questionnaires, for example, frequency of mitigations and exacerbations. Potential confounding factors included the psychological effects of living in this famous tourist city.

**Table 1:** Data collected from participants in the Sanya migratory cohort.

**Category**	**Data**
Demographics/socioeconomic factors	Date of birth, ethnicity, educational level, working status, sex, previous location of residency, and location and duration of current residency
Physical examination	Height, weight, BP, and lung function
Lifestyle	Diet, smoking, exercise, sleep, work, and living environments
Medical history	Allergic history, and family history of respiratory and allergic diseases
QoL evaluation	AQLQ and SGRQ

BP, Blood pressure; QoL, quality of life; AQLQ, Asthma Quality of Life Questionnaire; and SGRQ, Saint George Respiratory Questionnaire.

### Physical examination

We used a unified measuring tool for physical examination. After calibration, we measured the height, weight, and blood pressure of all participants, and in those with asthma and COPD, we also measured lung function. Participants were asked to take off their shoes and coats before their heights and weights were measured.

### Collection and measurement of biological samples

Nurses from local hospitals traveled to the community, collected 5–10 mL of patients' venous blood, and then transferred the samples to the laboratory at Sanya Women and Children's Hospital (SWCH) for centrifugation and subpackaging. Whole blood was used for routine blood tests; serum was used to test for liver and kidney function, blood sugar, blood lipids, and allergen. Some samples were stored in a freezer at −80°C. Table 2 lists the types of samples and main tests using these samples.

**Table 2:** Collection of biological samples from participants in the Sanya migratory cohort.

**Biological samples**	**Clinical examinations**	**Specific tests**
Venous blood	Routine blood	White blood cell (WBC), red blood cell (RBC), hemoglobin concentration (HGC), platelet count (PLT), neutrophil percentage (NEU%), lymphocyte percentage (LYM%), monocyte percentage (MPN%), eosinophil percentage (EOS%), and basophil percentage (BAS%)
	Liver function	Total protein (TP) level, albumin (ALB) level, globulin/white cell ratio (A/G), glutamic pyruvic transaminase (GPT), glutamic oxaloacetic transaminase (GOT), aspartate transaminase (AST)/alanine transaminase (ALT) ratio, total bilirubin (TBIL), direct bilirubin (DBIL), and indirect bilirubin (IBIL)
	Blood lipids and sugar	Glucose (Glu); total cholesterol (TC), triglyceride (TG), high-density lipoprotein cholesterol (HDL-C), low-density lipoprotein cholesterol (LDL-C), and blood glucose (BG)
	Allergens	Animal allergens (cat, horse, cow, and dog dander); house dust (household dust, dust mites, and cockroaches); food allergens (milk, eggs, soybeans, peanuts, cod, wheat, and millet); plant allergens (grass, French chrysanthemum, dandelion, plantain, *Chenopodium* [goosefoot], and a yellow flower); mycoallergens (*Penicillium punctatum, Aspergillus fumigatus, Polydendrosporium, Candida albicans, Alternaria alternata*, and *Helminthosporium longum*)
Urine	Routine urine	Environmental-pollution exposure

### Quality control

All investigators received unified training. Two investigators independently input the results of all questionnaires into database using EpiData 3.0 software (EpiData, Copenhagen, Demark). A Portion of the data underwent double entry, and in cases where inconsistencies arose, a third researcher reviewed and resolved them to guarantee data accuracy.

We adopted various policies to retain participants in the cohort. Health consultation was and will be provided during recruitment and follow-up, and free health examinations were and will be provided during baseline and follow-up. We are following up on participants in the spring (March–April) and autumn (October–December) every year for the next 3 years and providing them with timely reminders to take advantage of the free health examinations.

### Statistical analysis

Baseline characteristics of participants who stayed in or withdrew from the cohort, and loss of follow-up, were described. All missing data were noted. We analyzed classification data using a χ^2^ test and continuous data using Student's *t* test. All analyses were conducted using SPSS version 25.0 (IBM Corp., Armonk, NY, USA). *P* < 0.05 was considered to indicate a statistically significant difference.

### Ethical approval

The research protocol and informed-consent form of this study were reviewed and approved by the Ethics Committee of Sanya Women and Children's Hospital (Approval No. SYFYIRB2022009). All participants signed their informed consent before participating.

## Findings to date

### Baseline characteristics

The baseline survey was completed in March 2022 among the 756 volunteers who were recruited from the community (Table 3). After performing quality control, we excluded missing and unqualified participants, ultimately including a total of 693 in the cohort. These included 231 men (33.3%) and 462 women (66.7%), with an average age of 66.1 years. In terms of educational level, 26.7% have not completed junior high school, 32.1% have completed senior high school, and 37.3% are college graduates or undergraduates. Compared with their symptoms in their original places of residence, the initial rates of remission reported by the participants in Sanya were 80.4% for allergic rhinitis, 82.3% for bronchitis and emphysema, 85.2% for asthma, 96.0% for COPD (*P* < 0.001). The top five diseases in this cohort are allergic rhinitis (57.9%); eczema, urticaria, or dermatitis (35.6%); bronchitis and bronchiectasis (35.6%); asthma (14.7%); and emphysema (11.7%). The top five original regions of residence are Heilongjiang Province (40.9%), Liaoning Province (12.0%), Jilin Province (10.4%), Hebei Province (7.4%), and Beijing (6.9%). The first follow-up, of 682 participants, was conducted by telephone from October to December 2022. The loss of follow-up does not make sure since COVID-19.

**Table 3:** Baseline characteristics of the Sanya migratory cohort.

**Characteristics**	**Recruited for cohort (*N*)**	**Proportion (%) or Mean**
**Gender**		
Male	231	33.3
Female	462	66.7
Age (years)	693 (50–80)	66.1^*^
Height (cm)	671	162.0^*^
Weight (kg)	670	64.5^*^
BMI (kg/m^2^)	670	24.1^*^
**Educational level**		
Junior high school or lower	185	26.7
High school or technical secondary school	223	32.1
Undergraduate or junior college	258	37.3
Postgraduate or higher	9	1.3
**Respiratory/allergic disease**		
Asthma	102	14.7
COPD	77	10.6
Allergic rhinitis/pharyngitis	401	57.9
Eczema/urticaria/dermatitis	247	35.6
Food/drug allergy	60	8.7
Bronchitis/bronchiectasis	247	35.6
Emphysema	81	11.7
Hypertension	165	23.8
Diabetes	92	13.3
Other	51	7.4
**The original location of residence**		
Heilongjiang province	280	40.9
Jilin province	71	10.4
Liaoning province	82	12.0
Inner Mongolia Autonomous Region	40	5.8
Xinjiang Uygur Autonomous Region	21	3.1
Gansu province	6	0.9
Shanxi province	6	0.9
Beijing	48	6.9
Hebei province	51	7.4
Tianjin	10	1.4
Shaanxi province	26	3.8
Other	28	4.0
Sanya, > 5 years	15	2.2

BMI, Body mass index; COPD, chronic obstructive pulmonary disease; ^*^ Mean.

### Biological-sample collection

During the baseline survey, we collected venous blood for detection of allergen, routine blood, liver function, blood lipids and sugar, which is close linked to the allergic and immune diseases during March 2022; the collection rate was 100%. We have continued to collect venous blood and urine samples from all 693 participants for detection during the ongoing follow-up (Table 4).

**Table 4:** Data collected from participants in the Sanya migratory cohort.

**Participants' data**	**Baseline and follow-up**						
	**2022**		**2023**		**2024**		**2025**
	**Baseline**	**Autumn**	**Spring**	**Autumn**	**Spring**	**Autumn**	**Spring**
**Demographics/Social**							
Date of birth	**√**						
Ethnicity	**√**						
Educational level	**√**						
Marital status	**√**						
Gender	**√**						
The original location of residence	**√**						
Current location and duration of residence	**√**						
**Physical examination**							
Height	**√**	**√**	**√**	**√**	**√**	**√**	**√**
Weight	**√**	**√**	**√**	**√**	**√**	**√**	**√**
BP	**√**	**√**	**√**	**√**	**√**	**√**	**√**
Pulmonary function^#^		**√**	**√**	**√**	**√**	**√**	**√**
**Biological samples**							
Blood	**√**	**√**	**√**	**√**	**√**	**√**	**√**
Urine		**√**	**√**	**√**	**√**	**√**	**√**
**Lifestyle**							
Diet	**√**						**√**
Smoking/passive-smoking status	**√**						**√**
Exercise	**√**						**√**
Sleep habits	**√**						**√**
Living environment	**√**						**√**
**Medical records**							
Respiratory or allergic diseases	**√**						
Past allergic history, family history, and disease symptoms	**√**						
Medical history	**√**						
Family medical history	**√**						
Clinical test results	**√**	√	√	√	√	√	√
**Assessment of respiratory and allergic diseases**							
SGRQ	**√**		**√**		**√**		**√**
AQLQ	**√**		**√**		**√**		**√**

BP, blood pressure; ^#^for participants with COPD or asthma; SGRQ, Saint George Respiratory Questionnaire; and AQLQ, Asthma Quality of Life Questionnaire.

### Strengths and limitations

Sanya is located in the southernmost region of China, which is tropical and has a warm winter. Many MBEPs migrate from northern China to Sanya for the winter. This provides a unique opportunity to observe the effect of tropical climate and environment on allergic diseases and helps follow up with the general population. To the best of our knowledge, this is the world's first study on the effects of tropical climate and environmental factors on respiratory and allergic diseases in MBEPs. The biological samples collected will provide objective evidence of such effects. In the future, we will further explore the relationship between the climatic environment and allergic diseases in children.

However, this study has several limitations. The main disadvantage is that the volunteers recruited are migratory-bird middle-aged or old people, who have relatively high educational levels and socioeconomic status and are therefore not representative of the general population. This limits the generalization of our findings. In addition, MBEPs return to their original residences in spring, and the effect on participants living in northern regions during the spring and summer will be difficult to assess. Notably, such potential confounding variables were not adjusted for statistical adjustments, such as the psychological impact of living in a famous tourist city. If possible, we will perform further research into this aspect in the future.

## Collaboration

This is an ongoing prospective cohort study. Preliminary findings demonstrate the beneficial impact of tropical climate on allergic diseases in migratory-bird old people.

## Data availability statement

The data supporting the conclusions of this article will be made available by reasonable request.

## Ethics statement

The studies involving humans were approved by the Ethics Committee of Sanya Women and Children's Hospital (Approval No. SYFYIRB2022009). The studies were conducted in accordance with the local legislation and institutional requirements. Written informed consent for participation in this study was provided by the participants.

## Author contributions

HG: Data curation, Formal analysis, Investigation, Methodology, Project administration, Writing–original draft, Writing–review and editing. GY: Investigation, Methodology, Project administration, Resources, Writing—review and editing. JG: Investigation, Project administration, Resources, Writing—review and editing. XL: Formal analysis, Investigation, Project administration, Writing–review and editing. CL: Formal analysis, Investigation, Project administration, Writing—review and editing. HR: Data curation, Investigation, Writing–review and editing. DC: Investigation, Writing–review and editing. LZ: Investigation, Writing–review and editing. QH: Formal analysis, Investigation, Writing–review and editing. YH: Formal analysis, Investigation, Writing–review and editing. YZ: Project administration, Resources, Writing–review and editing. ST: Conceptualization, Methodology, Supervision, Writing–review and editing. ZL: Funding acquisition, Project administration, Resources, Writing–review and editing. SL: Conceptualization, Data curation, Formal analysis, Funding acquisition, Investigation, Methodology, Project administration, Resources, Supervision, Writing—original draft, Writing—review and editing. DW: Conceptualization, Formal analysis, Funding acquisition, Investigation, Methodology, Project administration, Resources, Writing—review and editing.

## References

[1] Brozek G Lawson J Szumilas D Zejda J. Increasing prevalence of asthma, respiratory symptoms, and allergic diseases: four repeated surveys from 1993-2014. Respir Med. (2015) 109:982–90. 10.1016/j.rmed.2015.05.010 26153339

[2] Biagioni B Annesi-Maesano I D'Amato G Cecchi L. The rising of allergic respiratory diseases in a changing world: from climate change to migration. Expert Rev Respir Med. (2020) 14:973–86. 10.1080/17476348.2020.1794829 32662693

[3] WHO. Asthma. World Health Organization (2022). Available online at: https://www.who.int/news-room/fact-sheets/detail/asthma (accessed October 20, 2022).

[4] Deng S-Z Jalaludin BB Antó JM Hess JJ Huang C-R. Climate change, air pollution, and allergic respiratory diseases: a call to action for health professionals. Chin Med J. (2020) 133:1552–60. 10.1097/CM9.0000000000000861 32590458 PMC7386356

[5] Brusselle GG Wai-San Ko F. Prevalence and burden of asthma in China: time to act. Lancet. (2019) 394:364–66. 10.1016/S0140-6736(19)31349-2 31230827

[6] Huang K Yang T Xu J Yang L Zhao J Zhang X. Prevalence, risk factors, and management of asthma in China: a national cross-sectional study. Lancet. (2019) 394:407–18. 10.1016/S0140-6736(19)31147-X 31230828

[7] Lin J Wang W Chen P Zhou X Wan H Yin K. Prevalence and risk factors of asthma in mainland China: the CARE study. Respir Med. (2018) 137:48–54. 10.1016/j.rmed.2018.02.010 29605212

[8] Wang XD Zheng M Lou HF Wang CS Zhang Y Bo MY. An increased prevalence of self-reported allergic rhinitis in major Chinese cities from 2005 to 2011. Allergy. (2016) 71:1170–80. 10.1111/all.12874 26948849 PMC5074323

[9] Yan S Wang X Yao Z Cheng J Ni H Xu Z. Seasonal characteristics of temperature variability impacts on childhood asthma hospitalization in Hefei, China: Does PM2. 5 modify the association? Environ Res. (2022) 207:112078. 10.1016/j.envres.2021.112078 34599899

[10] Aslam R Sharif F Baqar M Nizami A-S Ashraf U. Role of ambient air pollution in asthma spread among various population groups of Lahore City: a case study. Environ Sci Pollut Res Int. (2022) 30:8682–97. 10.1007/s11356-022-19086-1 35220536

[11] Schinasi LH Kenyon CC Hubbard RA Zhao Y Maltenfort M Melly SJ. Associations between high ambient temperatures and asthma exacerbation among children in Philadelphia, PA: a time series analysis. Occup Environ Med. (2022) 79:326–32. 10.1136/oemed-2021-107823 35246484

[12] Li M Chen S Zhao H Tang C Lai Y Ung COL. The short-term associations of chronic obstructive pulmonary disease hospitalizations with meteorological factors and air pollutants in Southwest China: a time-series study. Sci Rep. (2021) 11:12914. 10.1038/s41598-021-92380-z 34155257 PMC8217527

[13] Schoos AMM Chawes BL Bønnelykke K Stokholm J Rasmussen MA Bisgaard H. Increasing severity of early-onset atopic dermatitis, but not late-onset, associates with development of aeroallergen sensitization and allergic rhinitis in childhood. Allergy. (2022) 77:1254–62. 10.1111/all.15108 34558075

[14] Fieten KB Rijssenbeek-Nouwens LH Hashimoto S Bel EH Weersink EJ. Less exacerbations and sustained asthma control 12 months after high altitude climate treatment for severe asthma. Allergy. (2019) 74:628–30. 10.1111/all.13664 30428132

[15] Rijssenbeek-Nouwens LH Fieten KB Bron AO Hashimoto S Bel EH Weersink EJ. High-altitude treatment in atopic and nonatopic patients with severe asthma. Eur Respir J. (2012) 40:1374–80. 10.1183/09031936.00195211 22441741

[16] Wang P Wang J Zhang J Ma X Zhou L Sun Y. Spatial-temporal changes in ecosystem services and social-ecological drivers in a typical coastal tourism city: a case study of Sanya, China. Ecol Indic. (2022) 145:109607. 10.1016/j.ecolind.2022.109607

[17] Chen J Bao J. Chinese ‘snowbirds' in tropical Sanya: retirement migration and the production of translocal families. J Ethn Migr Stud. (2021) 47:2760–77. 10.1080/1369183X.2020.1739377

[18] Freidl J Huber D Braunschmid H Romodow C Pichler C Weisböck-Erdheim R. Winter exercise and speleotherapy for allergy and asthma: a randomized controlled clinical trial. J Clin Med. (2020) 9:3311. 10.3390/jcm9103311 33076411 PMC7602599

[19] Allahverdiyeva L Khalilova A Efendiyeva N Samedi V. Role of speleotherapy in complex treatment of pediatric asthma: single centre experience. J Allergy Clin Immunol. (2019) 143:AB104. 10.1016/j.jaci.2018.12.316

[20] Vinnikov D Khafagy A Blanc PD Brimkulov N Steinmaus C. High-altitude alpine therapy and lung function in asthma: systematic review and meta-analysis. ERJ Open Res. (2016) 2:48. 10.1183/13993003.congress-2016.PA4293 27730196 PMC5005180

[21] Leshem YA Chalmers JR Apfelbacher C Katoh N Gerbens LAA Schmitt J. Measuring atopic eczema control and itch intensity in clinical practice: a consensus statement from the harmonising outcome measures for eczema in clinical practice (HOME-CP) initiative. JAMA Dermatol. (2022) 158:1429–35. 10.1001/jamadermatol.2022.4211 36223090

[22] Ellwood P Asher MI Björkstén B Burr M Pearce N Robertson CF. Diet and asthma, allergic rhinoconjunctivitis and atopic eczema symptom prevalence: an ecological analysis of the international study of asthma and allergies in childhood (ISAAC) data. ISAAC phase one study group. Eur Respir J. (2001) 17:436–43. 10.1183/09031936.01.17304360 11405522

[23] Vogelmeier CF Criner GJ Martinez FJ Anzueto A Barnes PJ Bourbeau J. Global strategy for the diagnosis, management, and prevention of chronic obstructive lung disease 2017 report. GOLD executive summary. Am J Respir Crit Care Med. (2017) 195:557–82. 10.1164/rccm.201701-0218PP 28128970

[24] Miele CH Jaganath D Miranda JJ Bernabe-Ortiz A Gilman RH Johnson CM. Urbanization and daily exposure to biomass fuel smoke both contribute to chronic bronchitis risk in a population with low prevalence of daily tobacco smoking. Copd. (2016) 13:186–95. 10.3109/15412555.2015.1067765 26552585 PMC4955773

[25] Khusial RJ Honkoop PJ van der Meer V Snoeck-Stroband JB Sont JK. Validation of online asthma control questionnaire and asthma quality of life questionnaire. ERJ Open Res. (2020) 6:00289–2019. 10.1183/23120541.00289-2019 32010723 PMC6983500

[26] Zhu B Wang Y Ming J Chen W Zhang L. Disease burden of COPD in China: a systematic review. Int J Chron Obstruct Pulmon Dis. (2018) 13:1353. 10.2147/COPD.S161555 29731623 PMC5927339

